# A Rare Case of Obstructed Recurrent Incisional Hernia With Incidentalomas

**DOI:** 10.7759/cureus.53473

**Published:** 2024-02-02

**Authors:** Tapesh D Nagaria, Raju K Shinde, Samarth Shukla, Sourya Acharya, Neema Acharya, Sajika P Dighe

**Affiliations:** 1 General Surgery, Jawaharlal Nehru Medical College, Datta Meghe Institute of Higher Education and Research, Wardha, IND; 2 Pathology, Jawaharlal Nehru Medical College, Datta Meghe Institute of Higher Education and Research, Wardha, IND; 3 Medicine, Jawaharlal Nehru Medical College, Datta Meghe Institute of Higher Education and Research, Wardha, IND; 4 Obstetrics and Gynaecology, Jawaharlal Nehru Medical College, Datta Meghe Institute of Higher Education and Research, Wardha, IND; 5 General Surgery, Lokmanya Tilak Municipal Medical College, Mumbai, IND

**Keywords:** obstruction, incisional hernia, computed tomography, surgery, adrenal adenoma

## Abstract

Incisional hernias (IHs) are the most common postoperative complication of incisions during laparotomy and contribute to a significant burden. The aetiology of IHs varies depending on the surgical technique, patient's condition, and surgeon's experience. Many patients present with abdominal swelling and some degree of discomfort, and in an emergency, the presentation is usually as bowel obstruction or strangulation, necessitating immediate exploration. Hernias can be repaired by closing the defect with a nonabsorbable suture or using mesh. Amidst the use of invasive techniques and mesh, the rate of recurrence remains high for IHs, with pain and infection being the most common symptoms. The consequence of IH repair is affected by comorbid conditions such as chronic cough, constipation, urethral stricture, benign prostate hyperplasia, ascites, and obesity. We present a case of a 63-year-old male with an IH, adrenal adenoma, and adrenal cyst, which was an incidental finding.

## Introduction

Incisional hernia (IH) arises from the abdominal wall, which develops at the site of an earlier surgical incision. Midline IHs are the most common ones. Patients with IH are also at risk of incarceration, bowel obstruction, and strangulation. Wound healing issues are estimated to occur in at least 20% of laparotomy cases, with many resulting in an IH [1]. At first, the hernia may not be visible. However, it advances in almost all cases, and the patient complains of a bulge. The condition can affect both males and females of all ages [1].

IH has been reported in the aftermath of traumatic abdominal wall injuries. It happens when the abdominal wall does not close on its own properly after surgery, either due to technical factors or due to patient-related factors. Despite major advances in closure techniques of the abdominal wall, the rate of IH post-laparotomy can range from 15 to 20%. Despite ongoing studies for the best closure methods to prevent IH and the publication of current reports, IH continues to be a problem for surgeons [2-4]. Surgical repair or nonoperative treatment are options for an IH [5].

IH causes discomfort as they grow, limiting patients' ability to work and participate in other physical activities. Cosmetic issues may also arise. Overall, a patient's quality of life can be greatly impacted. IH complications include pain, bowel obstruction, incarceration, and strangulation, as well as the risk of needing to undergo repeat surgery. IHs are commonly repaired using open, laparoscopic, and robotic techniques customized according to the patient and hernia characteristics [6]. IH repair has also been linked to hernia recurrence rates ranging from 10 to 50%, as well as significant mortality and morbidity. The recurrence rate of hernia remains relatively constant over time as surgeons continue to encounter increasingly remarkable patient factors such as comorbidity, old age, and more obese patients undergoing primary surgery [7].

This report presents a unique case of occurrence of adrenal adenoma and adrenal cyst, which are incidental findings with the chief finding of IH.

## Case presentation

A 63-year-old male presented to the emergency room, with a history of constipation for three days, with multiple episodes of vomiting in the last three days and breathlessness. The patient gave a history of an abdominal open surgery performed 25 years prior to presentation. Still, the exact nature of the procedure and disease could not be ascertained due to the unavailability of documents. The patient also underwent an infra umbilical IH meshplasty repair seven years prior to presentation. The patient gave a history of cigarette smoking and of being diagnosed with diabetes mellitus in the past but discontinued medications. On examination, the patient was found to have an infra umbilical irreducible IH with bowel as content.

Rectal examination revealed a stool-loaded rectum and manual removal of feces was done. Blood investigations revealed a raised white blood cell (WBC) count at 19,100/mm^3^, mildly raised serum creatinine at 1.6 mg/dl, raised serum HbA1C at 7.6 mg/dl, raised C-reactive protein at 40 mg/dl, and albumin at 3.8 g/dl.

**Figure 1:**
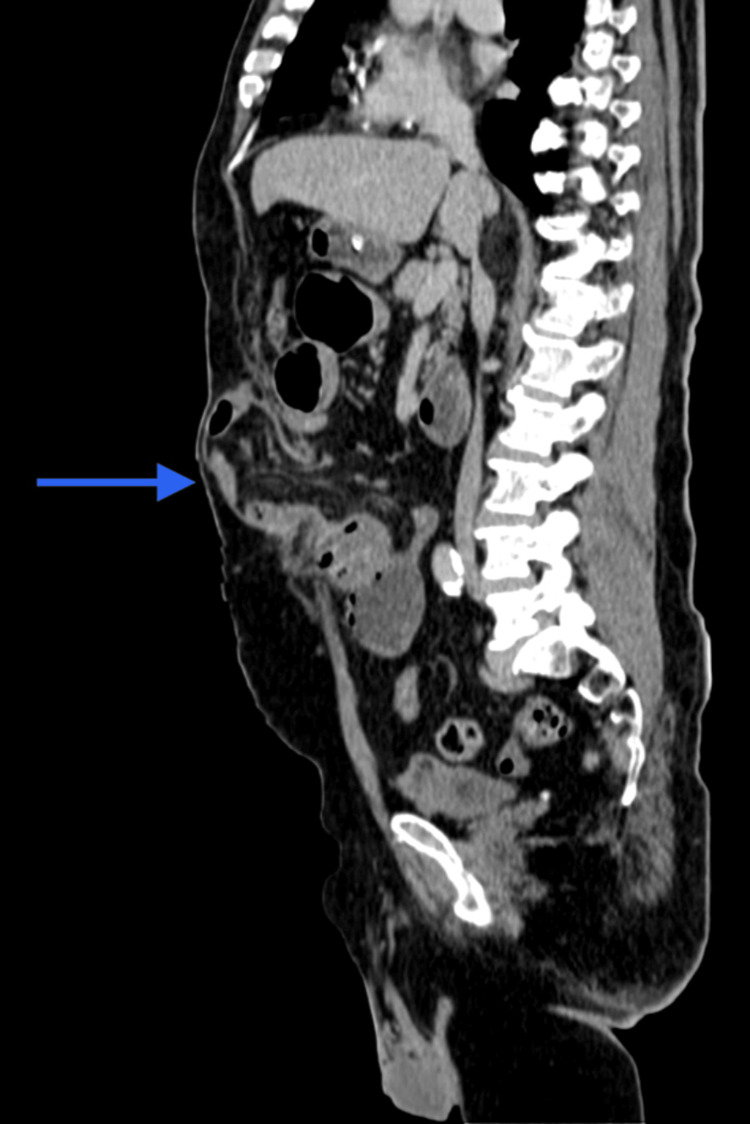
CECT suggests an anterior abdominal wall defect of size 4.6 x 4.8 cm at the level of L1-L2. CECT: Contract-enhanced computed tomography

A contract-enhanced computed tomography (CECT) was done and was suggestive of an anterior abdominal wall defect of size 4.6 x 4.8 cm at the level of L1-L2, with herniation of jejunal and ileal bowel loops and mesentery and fat stranding albeit, with uniform mucosal enhancement in the arterial phase (Figure 1).

**Figure 2:**
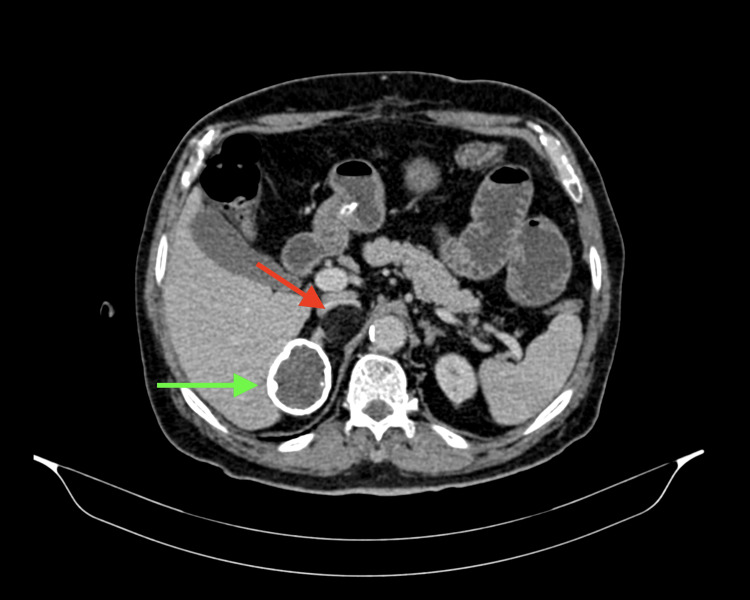
CECT shows lipid-rich adrenal adenoma (red arrow) and calcified margins in the right suprarenal region of size 5 x 4 cm (green arrow). CECT: Contract-enhanced computed tomography

CECT also revealed two well-defined fat density lesions, the largest measuring 6.2 x 3.8 cm in the retroperitoneum arising from the right adrenal and indenting over the inferior vena cava posteriorly, likely to be a lipid-rich adrenal adenoma. Another incidental finding was a well-defined, non-enhancing mixed-density lesion with calcified margins in the right suprarenal region of size 5 x 4 cm, likely to be a calcified adrenal cyst (Figure 2).

**Figure 3:**
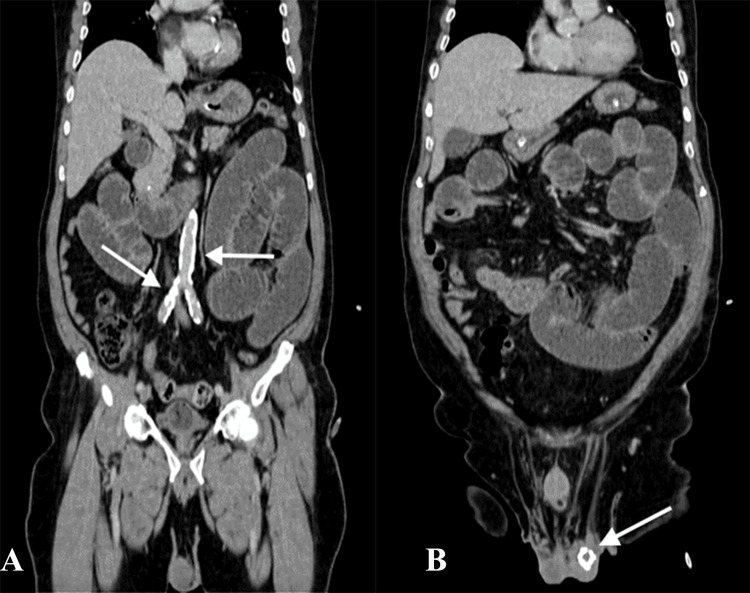
Image A shows aortic and iliac calcification; B shows a calcified left epididymal cyst of size 1.9 x 1.7 cm.

CECT also showed extensive vascular calcifications along the aorta and the bilateral common iliac vessels (Figure 3A) and a calcified left epididymal cyst of size 1.9 x 1.7 cm (Figure 3B).

As the patient was in obstruction, after the induction of general anaesthesia, an incision was taken around the previous scar of approximately 12 cm. The previous scar tissue was excised and the incision was deepened in layers. Multiple dense adhesions were noted between the rectus sheath and peritoneum. A defect of approximately 5 cm was noted on the right side and 10 cm on the left side with a sac containing jejunal and ilial loops. Adhesions were found between bowel loops and hernia sac. Adhesiolysis was done, freeing bowel loops from the sac. Bowel loops reduced from the sac and found viable with good peristalsis. Mesh from the previous surgery was found in the retrorectus space and excised. A warm saline wash was given. Closure done en masse with Prolene 1 (Ethicon, Inc., New Jersey, United States) on loop. Skin closure was done with Ethilon 2-0 RC (Ethicon, Inc.). Sterile compression dressing was done and the patient was shifted out in an intubated state.

Five days post surgery, the patient developed mild hypertension, which was managed with supportive care. The patient recovered well after the procedure and was discharged.

## Discussion

IH is a common complication of open laparotomies and abdominal surgeries and is a source of significant morbidity and mortality in patients, especially those with significant comorbidities. Recurrence rates vary between as high as 49% in cases of open suture repair of IH and 10% in cases of open mesh repair [8]. Small bowel obstruction post-recurrence is a known complication of open IH repair, possibly due to the presence of adhesions.

Adrenal adenomas, also known as adrenal incidentalomas, are benign, mostly non-functional neoplasms arising from the adrenal cortex and are discovered incidentally during imaging techniques. Functional adrenal adenomas are known to produce adrenal cortical hormones. On CT scans, the prevalence of adrenal incidentalomas ranges from 0.35 to 1.9% [9]. Adrenal adenomas are found in approximately 54% of them. Adrenal adenomas are associated with a high degree of occurrence of adverse cardiovascular events as well as the presence of atherosclerosis, hypertension, and diabetes mellitus, as seen in this patient [10]. The vascular calcification in the aorta and the common iliac arteries are common findings, usually subclinical, in males above 60 years of age [11] and may be a metabolic consequence of the adrenal incidentaloma secreting small amounts of glucocorticoids [12]. Adrenal cystic lesions are an uncommon occurrence per se and are discovered incidentally or sometimes are symptomatic. They are broadly classified into endothelial cysts, pseudocysts, epithelial cysts, and parasitic cysts. Calcified adrenal cysts are frequently associated with adrenal cortical adenomas and carcinomas and rarely with pheochromocytomas [13]. Management of adrenal incidentalomas if asymptomatic is usually to observe, and if symptomatic, requires complete tumour workup. In our case also, the patient had non-functional incidentolomas, not causing any clinical symptoms. Calcified epididymal cysts are also a rare presentation, although an incidental finding and not associated with the other lesions [14].

## Conclusions

We presented a rare case of a 63-year-old patient having obstructed IHs, which are fairly common and were treated successfully. Multiple incidental findings of adrenal adenoma with a large calcified adrenal cyst and extensive atherosclerosis with calcification through the aorta and common iliac vessels were rare findings found in this patient. This case is rare because multiple lesions existed together, that is, adenoma and adrenal cyst, which were incidental findings and found to be non-functional. Thus, it was left untreated due to the unwillingness of the patient and did not have any overt clinical features.

It is crucial to perform all the required examinations of the patient carefully to prevent misdiagnosis and manage the incidental findings accordingly, especially if they are functional and present with clinical symptoms, as the functional incidentalomas can interfere with the recovery of the patient treated for the main presenting cause.
